# Osteoprotegerina: Um Biomarcador na Doença Renal Crônica

**DOI:** 10.36660/abc.20240679

**Published:** 2024-11-12

**Authors:** Rui Póvoa

**Affiliations:** 1 Universidade Federal de São Paulo Setor de Cardiopatia Hipertensiva São Paulo SP Brasil Setor de Cardiopatia Hipertensiva da Universidade Federal de São Paulo, São Paulo, SP – Brasil; 2 Universidade Federal de São Paulo São Paulo SP Brasil Disciplina de Cardiologia da Universidade Federal de São Paulo, São Paulo, SP – Brasil

**Keywords:** Biomarcadores, Osteoprotegerina, Calcificação Vascular, Doença Renal Crônica

Os biomarcadores são indicadores biológicos que podem ser quantificados para avaliar processos biológicos normais ou patológicos e respostas às diversas intervenções terapêuticas.^1^ São encontrados nos diversos fluidos corporais, tais como sangue, urina ou em tecidos e ajudam no diagnóstico precoce de algumas doenças, além do monitoramento da progressão ou da avaliação da eficácia do tratamento. Além da importância no diagnóstico clínico, podem estimar o risco e o prognóstico. Isto é muito bem estruturado nos processos cuja base inflamatória se destaca em um cenário multifatorial, tais como a hipertensão arterial, diabetes mellitus, dislipidemias e doença renal crônica (DRC).^2,3^

A DRC, que é um problema crescente de saúde pública, cada vez mais presente no cenário das doenças cardiovasculares (CVs) devido à alta morbimortalidade. Está no final evolutivo da sequência do *continuum* CV onde os principais fatores de risco (hipertensão, diabetes, dislipidemia) não foram tratados de forma adequada. Além disso, os pacientes com DRC estão ainda sob um risco CV maior devido à presença destes fatores que muitas vezes continuam sem tratamento adequado.^4^

Na medicina de precisão é necessária para a identificação e a quantificação de diversos elementos dependendo dos aspectos clínicos e da proposta terapêutica em cada doença de forma particular.^5^ Na DRC a utilização de potenciais biomarcadores otimiza a medicina de precisão, podendo fornecer uma nova estratégia para conhecer os meandros fisiopatológicos da evolução deste complexo mecanismo de perda evolutiva da função renal.^6^

Assim, a osteoprotegerina (OPG), uma glicoproteína pertencente ao grupo dos receptores do fator de necrose tumoral, participa na regulação do metabolismo ósseo e tem sido associada à calcificação vascular e à doença arterial coronária, estando seus níveis associados com maior incidência e prevalência de doenças CVs.^7^

O trabalho de Matos TO et al. avaliou em pacientes com função renal diminuída em estágio 3 (TFG <60 ml/min/1,73 m^2^), a relação da OPC com diversos parâmetros CVs que se relacionam com o risco CV ou com a função endotelial.^8^ O racional foi estudar a relação da OPG com a DRC e analisar diversos parâmetros CVs, visto a relação deste biomarcador com o metabolismo do cálcio na matriz vascular e a consequente calcificação. Geralmente os níveis de OPG estão elevados nas fases terminais da DRC e é um forte preditor de calcificação vascular em todos os estágios da disfunção renal.

Os resultados foram interessantes, mostrando a relação positiva e significante deste biomarcador do metabolismo ósseo, com os parâmetros hemodinâmicos da pressão de pulso central, velocidade de onda de pulso (VOP), *augmentation index*, pressão arterial sistólica periférica e pressão arterial sistólica central. Todos são fatores de risco independentes para a maioria das doenças CVs.

A prevalência da calcificação vascular e da calcificação das artérias coronárias é frequente na DRC, principalmente nas fases mais avançadas. Porém podem se instalar nas fases mais precoces e são um fator de risco independente para as doenças CV.^9^ Por isso, a importância deste biomarcador é a detecção precoce da deposição de cálcio nas artérias.^10^

Deve-se destacar a correlação positiva e significante da OPG com a VOP, que se correlaciona com a idade vascular, refletindo do caso da DRC a presença de lesão ou envelhecimento vascular mais precoce. Entretanto a dilatação mediada pelo fluxo não apresentou qualquer relação com a OPG. Sabemos que é utilizada para avaliar a função endotelial, porém estas alterações endoteliais na DRC ainda não têm os aspectos fisiopatológicos bem definidos. Diversos mecanismos podem estar envolvidos ou participar de forma peculiar alterando a função endotelial e não somente o metabolismo do óxido nítrico. A própria calcificação vascular está envolvida globalmente sendo o destaque nesta disfunção.^11^

Neste trabalho de Matos et al. os níveis de OPG tiveram uma relação direta e crescente com o grau da DRC, mostrando uma relação tipo "dose/efeito" aumentando a sua validade como marcador.^8^ Ainda não sabemos de que forma a OPG está envolvida na patogenia da calcificação da arterial, os meandros fisiopatológicos no contexto do metabolismo do cálcio e a verdadeira relação com o prognóstico da doença renal.^12^

Este estudo pioneiro na população brasileira deu os primeiros passos para compreendermos o valor de determinados biomarcadores, e sua relação diagnóstica e prognóstica na doença renal onde a esfera de morbimortalidade é tão grave e não só explicada pelos fatores de risco clássicos comuns às doenças CV.
