# Supplementary material for: Osteoprotegerina: Um Biomarcador na Doença Renal Crônica
Source: Arq Bras Cardiol. 2024 Nov 12;121(11):e20240679. [Article in Portuguese] doi: 10.36660/abc.20240679 (PMC11634296; doi:10.36660/abc.20240679)
Supplement: Translation

# Translation: Osteoprotegerin: A Biomarker in Chronic Kidney Disease

**DOI:** 10.36660/abc.20240679i

**Authors:** Rui Póvoa

**Affiliations:** 1 São Paulo SP Brazil Setor de Cardiopatia Hipertensiva da Universidade Federal de São Paulo, São Paulo, SP – Brazil; 2 São Paulo SP Brazil Disciplina de Cardiologia da Universidade Federal de São Paulo, São Paulo, SP – Brazil

**Keywords:** Biomarkers, Osteoprotegerin, Vascular Calcification, Chronic Kidney Disease

Biomarkers are biological indicators that can be quantified to assess normal or pathological biological processes and responses to various therapeutic interventions.^1^ They are found in various body fluids, such as blood, urine, or tissues, and help in the early diagnosis of some diseases, in addition to monitoring progression or evaluating the effectiveness of treatment. In addition to their importance in clinical diagnosis, they can estimate risk and prognosis. This is very well structured in processes whose inflammatory basis stands out in a multifactorial scenario, such as arterial hypertension, diabetes mellitus, dyslipidemia, and chronic kidney disease (CKD).^2,3^

CKD, which is a growing public health problem, is increasingly present in the cardiovascular (CV) disease scenario due to its high morbidity and mortality. It is at the evolutionary end of the CV continuum where the main risk factors (hypertension, diabetes, dyslipidemia) have not been adequately treated. In addition, patients with CKD are still at a higher CV risk due to the presence of these factors that often remain without adequate treatment.^4^

In precision medicine, it is necessary to identify and quantify several elements depending on the clinical aspects and the therapeutic proposal for each disease in a particular way.^5^ In CKD, the use of potential biomarkers optimizes precision medicine and can provide a new strategy to understand the pathophysiological intricacies of the evolution of this complex mechanism of progressive loss of renal function.^6^

Thus, osteoprotegerin (OPG), a glycoprotein belonging to the group of tumor necrosis factor receptors, participates in the regulation of bone metabolism and has been associated with vascular calcification and coronary artery disease, with its levels being associated with a higher incidence and prevalence of CV diseases. ^7^

The study by Matos TO et al. evaluated the relationship between OPG and several CV parameters related to CV risk or endothelial function in patients with stage 3 renal function impairment (GFR <60 ml/min/1.73 m^2^).^8^ The rationale was to study the relationship between OPG and CKD and to analyze several CV parameters, given the relationship of this biomarker with calcium metabolism in the vascular matrix and consequent calcification. OPG levels are generally elevated in the terminal stages of CKD and are a strong predictor of vascular calcification in all stages of renal dysfunction.

The results were interesting, showing the positive and significant relationship of this bone metabolism biomarker with the hemodynamic parameters of central pulse pressure, pulse wave velocity (PWV), augmentation index, peripheral systolic blood pressure, and central systolic blood pressure. All of these are independent risk factors for most CV diseases.

The prevalence of vascular calcification and coronary artery calcification is common in CKD, especially in the more advanced stages. However, they can occur in the earlier stages and are an independent risk factor for CV disease.^9^ Therefore, the importance of this biomarker is the early detection of calcium deposition in the arteries.^10^

It is worth highlighting the positive and significant correlation between OPG and PWV, which correlates with vascular age, reflecting in the case of CKD the presence of injury or earlier vascular aging. However, flow-mediated dilation did not show any relationship with OPG. We know that it is used to assess endothelial function, but the pathophysiological aspects of these endothelial changes in CKD do not yet have well-defined pathophysiological aspects. Several mechanisms may be involved or participate peculiarly, altering endothelial function and not only nitric oxide metabolism. Vascular calcification itself is globally engaged and is the highlight of this dysfunction.^11^

In this work by Matos et al., OPG levels had a direct and increasing relationship with the degree of CKD, showing a "dose/effect" relationship, increasing its validity as a marker.^8^ We still do not know how OPG is involved in the pathogenesis of arterial calcification, the pathophysiological intricacies in the context of calcium metabolism, and the true relationship with the prognosis of kidney disease.^12^

This pioneering study in the Brazilian population took the first steps towards understanding the value of certain biomarkers and their diagnostic and prognostic relationship in kidney disease, where the sphere of morbidity and mortality is so serious and not only explained by the classic risk factors common to CV diseases.
